# Propolis Extract as Antioxidant to Improve Oxidative Stability of Fresh Patties during Refrigerated Storage

**DOI:** 10.3390/foods8120614

**Published:** 2019-11-24

**Authors:** Rey David Vargas-Sánchez, Gastón Ramón Torrescano-Urrutia, Brisa del Mar Torres-Martínez, Mirian Pateiro, José Manuel Lorenzo, Armida Sánchez-Escalante

**Affiliations:** 1 Coordinación de Tecnología de Alimentos de Origen Animal (CTAOA), Centro de Investigación en Alimentación y Desarrollo, A.C. (CIAD), Carretera Gustavo Enrique Astiazarán Rosas, 46, Hermosillo, Sonora 83303, Mexico; rey.vargas@ciad.mx (R.D.V.-S.); gtorrescano@ciad.mx (G.R.T.-U.); brisa.torres@estudiantes.ciad.mx (B.d.M.T.-M.); 2 Centro Tecnológico de la Carne de Galicia, Rúa Galicia No. 4, Parque Tecnológico de Galicia, San Cibrao das Viñas, 32900 Ourense, Spain; jmlorenzo@ceteca.net

**Keywords:** propolis, ethanol extract, antioxidant, meat quality

## Abstract

The effect of propolis ethanol extract (PEE), butylated hydroxytoluene (BHT), and ascorbic acid (Asc) against lipid (Lox) and protein oxidation (Pox), color deterioration, and the antioxidant stabilizer of raw beef and pork patties during chilled storage (9 days at 2 °C/under darkness) was investigated. Total phenolic content (TPC), reducing power ability (RPA), DPPH^●^ radical scavenging activity (FRSA) of the PEE was evaluated. Meat samples were evaluated for pH, Lox (TBARS), Pox (Carbonyls), color (L*, a*, b*, C*, and h*), metmyoglobin formation (MMb), TPC, RPA, and FRSA. Results indicated that PEE is rich in phenolic content and antioxidant activity, and their incorporation in beef and pork patties reduced (*p* < 0.05) Lox and Pox (TBARS-88.7 and 80% inhibition; Pox-47.3 and 30.6% inhibition, respectively), as well as loss of color and increased the oxidative stability throughout storage.

## 1. Introduction

Meat is an important source of dietary nutrients for human metabolic processes such as lipids, proteins, vitamins, and minerals [1]. Lipids provide indispensable dietary energy and essential nutrients such as essential fatty acids, i.e., mono and polyunsaturated fatty acids (MUFA and PUFA, respectively), while meat protein is distinguished due to its richness in all the essential amino acids [1,2]. However, the secondary metabolites derived from lipid and protein oxidation (Lox and Pox, respectively) are considered causes of quality loss during the processing and storage of meat and meat products and may result in negative effects on sensory and processing properties [3,4]. Thus, synthetic antioxidants have been widely used in the meat industry to reduce Lox and Pox process. Nevertheless, the use of synthetic antioxidants, such as propyl gallate (PG), tert-butylhydroquinone (TBHQ), butylated hydroxyanisole (BHA) and butylated hydroxytoluene (BHT), have shown potential health risks [5].

Therefore, there have been efforts to obtain natural antioxidants capable of preserving meat and meat products against undesirable changes caused by oxidation processes [6,7,8]. Based on their high phenolic composition, natural antioxidant sources, such as fruits (apple, bearberry, blueberry, cranberry, pomegranate, strawberry, tomato, among others), vegetables (broccoli, carrot, pumpkin, among others), herbs and spices (black and red pepper, cinnamon, clove, coriander, green tea, moringa, olive, onion, oregano, rosemary, sage, sesame, turmeric, among others) and honeybee products (honey, royal jelly, pollen, and propolis), have shown to decrease oxidation as effectively as synthetic antioxidant [6,9,10,11,12,13,14].

Propolis is a substance of complex composition and viscous consistency that bees (*Apis mellifera*) collect from resinous and pollen material from different trees and plants, moistened with saliva and enzymatic secretions, and mixed with the wax produced by the wax glands. In addition, it has been demonstrated that propolis extracts exhibit potential health effects, such as anti-inflammatory, antihypertensive, antidiabetic, antimicrobial, and antioxidant activity, which could be affected by the botanical source, season of collection, and phenolic composition [15,16]. Additionally, it has been proposed as an ingredient to increase the shelf life of fruits, vegetable oils, and meat and dairy products [17,18]. Its use as an antioxidant in meat products allowed the reduction of lipid oxidation [19], avoiding the reduction of sensory quality of products associated with changes in color, texture, and appearance of rancid odor and flavor, which influences consumer acceptance [20], as well as a decrease in the nutritional value due to the loss of essential fatty acids and vitamins [4]. In this way, a preliminary study carried out by our research group showed that propolis extract (PE, 2%) incorporation in bovine and porcine patties significantly reduced (*p* < 0.05) Lox and Pox, as well as color loss, thereby increasing the oxidative stability of patties during refrigerated storage [21].

In addition, novel technologies for the application of propolis extract in meat products, such as its inclusion in packaging materials and the microencapsulation, have been evaluated [22]. In this regard, a recent study evaluated the effect of microencapsulated propolis co-product extract (MPC) on oxidative stability of rear lean beef burgers during frozen storage [23]. The MPC addition inhibited lipid oxidation and did not affect color, appearance, and texture of beef burgers, even improving the results obtained with the synthetic antioxidant. This technique was also applied by other authors in an Italian-type salami, reducing some strong sensorial characteristics associated with the use of propolis [24].

However, data on the effect of propolis addition on beef and pork burgers during cold storage are still limited. Therefore, in this work, the effect of propolis extract on Lox, Pox, color changes, metmyoglobin formation, pH changes, total phenolic content, free radical scavenging activity, and the reducing power ability of beef and pork patties in comparison to ascorbic acid and butylhydroxytoluene addition was evaluated during cold storage.

## 2. Materials and Methods

### 2.1. Chemicals and Reagents

All the chemical products used were of analytical grade. Folin-Ciocalteu’s reagent, sodium carbonate, sodium phosphate, potassium ferricyanide, iron chloride, 1,1-diphenyl-2-picrylhydrazyl (DPPH^●^), ethanol and methanol (HPLC), formic acid, hexane, 2-propanol, butylated hydroxytoluene, ascorbic acid, gallic acid, cinnamic acid, *p*-coumaric acid, ferulic acid, acacetin, apigenin, CAPE (caffeic acid phenetyl esther), chrysin, galangin, luteolin, and quercetin were purchased from Sigma Chemicals (St. Louis, MO, USA). While, 2-thiobarbituric acid and trichloroacetic acid were obtained from J.T. Baker^®^. Naringenin, kaempferol, pinocembrin, pinostrobin, and pinobaksin 3-acetate were purchased from INDOFINE (Chemical Company Inc., Hillsborough, NJ, USA).

### 2.2. Propolis Ethanol Extract Preparation (PEE)

Propolis samples used in this study were collected from the Northwest of Mexico (29.1476 N, -110.1239 O; 632 m). The location presented desert climate characteristics, and the sampling site is surrounded by foothills or thornscrub, dominated by *Fabaceae* species (*Prosopis velutina* and *Mimosa distachya*). Then, phenolic compounds of raw propolis were extracted with ethanol (1:10) at room temperature (25 °C) for 3 days, filtered (Whatman No. 4 filter paper), and concentrated under reduced pressure (Rotary evaporator BÜCHI R-200, Flawil, Switzerland), washed with hexane to remove waxes, and stored under dark at −20 °C, until analysis [25].

### 2.3. Phenolic and Antioxidant Activity of PEE

#### 2.3.1. Total Phenolic Content

Total phenolic content (TPC) of PEE was determined according to the Folin-Ciocalteu’s procedure [26]. PEE (100 µL, at different concentrations) was oxidized with 250 µL of Folin-Ciocalteu´s reagent (2 M). The mixture was homogenized and incubated for 8 min, under dark conditions. Then, 750 µL of Na_2_CO_3_ (7%, w/v) were added. The reaction mixture was incubated for 30 min, under dark conditions. The absorbance was measured at 765 nm in a spectrophotometer (Model 336001, Spectronic Genesys 5, Thermo Electron Corp., Madison, WI, USA). The results were expressed as mg of gallic acid equivalents/g of extract (mg GAE/g).

#### 2.3.2. HPLC-DAD Analysis

The phenolic compounds in PEE were identified using an HPLC system (Varian ProStar, Walnut Creek, CA, USA) equipped with a diode array detector (DAD). The stationary phase was a C18 LiChrospher 5 column (125 × 4.0 mm, 5 mm). The flow rate was 1 mL/min using 5% formic acid in water and methanol as the elution solvent (solvent A and B, respectively). The gradient program profile was as follows: starting with 0% B (0 min), 30% (10–20 min), 40% (20–30 min), 45% (30–50 min), 60% (50–52 min), 80% (52–65 min), 100% (65–70 min), and 0% (70–71 min). The elution of the compounds was monitored at 280 and 340 nm. The assignation of peaks was performed comparing the retention times using authentic standards solutions and by spiking the samples with the respective compounds. The calibration curves were prepared using each compound (15 to 500 µg/mL) for quantification, and linear ranges were determined *r* ≥ 0.99 [25].

#### 2.3.3. Reducing-Power Assay

Reducing-power ability (RPA) was determined by the Prussian blue method [27]. PEE (100 µL, at different concentrations) was homogenized with 300 µL of phosphate buffer (0.2 M, pH 6.6), 300 µL of 1% potassium ferric cyanide and incubated in a water bath at 50 °C for 20 min. After, 300 µL of 10% trichloroacetic acid (TCA) were added and centrifuged at 4200× *g* for 10 min. The supernatant (500 µL) was mixed with 100 µL of distilled water and 250 µL of 0.1% ferric chloride. The absorbance was measured at 700 nm. The results were expressed as absorbance increase at the same wavelength.

#### 2.3.4. Free-Radical Scavenging Activity

The DPPH^●^ radical-scavenging activity (FRSA) was determined [28]. The PEE (500 µL) were homogenized with 500 µL of DPPH^●^ ethanol solution (300 µM) and incubated at room temperature for 30 min. The absorbance was measured at 517 nm. FRSA was calculated as (Ac–As) × 100/Ac, where Ac is the absorbance of the control (*t* = 0 min), and As is the absorbance of the sample (*t* = 30 min).

### 2.4. Beef and Pork Patties Manufacture

The patties were prepared, as described previously [29]. Fresh beef and pork minced meat were obtained from *Semimembranosus* muscle after 48 h *postmortem* at a local processor and mixed in separate batches with 1.5% salt (NaCl, w/w), and 10% of beef and pork back fat, respectively, in the final formulation (w/w). A total of 24 patties (25 g/patty) per treatment were formed and placed on a Styrofoam™ tray. The polystyrene trays with patties were wrapped with polyvinyl chloride film (17,400 cm^3^ O_2_/m^2^/24 h at 23 °C). The patties were subjected to refrigerated storage at 2 °C under dark for 0, 3, 6, and 9 days, and 2 packs were opened for subsequent analysis for each formulation. In each replication (twice), beef and pork patties were assessed in eight treatments as follows: (1) B (negative control, beef patties without antioxidant); (2) B+PEE (beef patties with 2% of PEE, w/w); (3) B+BHT (positive control, beef patties with 0.02% of BHT, fat basis w/w); (4) B+Asc (positive control, beef patties with 0.015% of Asc, w/w); (5) P (negative control, porcine patties without antioxidant); (6) P+PEE (porcine patties with 2% of PEE, w/w); (7) P+BHT (positive control, porcine patties with 0.02% of BHT, w/w); (8) P+Asc (positive control, porcine patties with 0.015% of Asc, w/w).

### 2.5. Phenolic and Antioxidant Activity of Meat Extract

Phenolic and antioxidant activity of meat extract was assayed by TPC, RPA, and FRSA methods. The meat extract was obtained homogenizing beef and pork patties with ethanol (1:10, w/v) at 4200× *g* for 10 min at 4 °C. Then, the resulting supernatant was employed to carry out the aforementioned measurements [30].

### 2.6. pH and Color Changes of Patties

#### 2.6.1. pH Measurement

Meat samples were homogenized with distilled water (1:10, w/v) at 4500 rpm for 1 min (Ultra-Turrax model T25, IKA^®^, Staufen, Germany) in an ice bath. pH values were measured with a potentiometer equipped with a glass electrode and automatic compensation of temperature (Model pH211, Hanna Instruments Inc., Woonsocket, RI, USA.) [31].

#### 2.6.2. Color Measurement

The color changes were measured using a spectrophotometer (model CM 508d, Konica Minolta Inc., Tokyo, Japan). The values registered were lightness (L∗), redness (a∗), yellowness (b∗), chromaticity (C∗), and hue angle (h∗). The beef and pork patties were extracted from their packaging and exposed at 4 °C for 30 min. In total, 10 measurements were performed on the surface of each sample [32].

#### 2.6.3. Metmyoglobin Formation

The metmyoglobin formation (MMb) was determined spectrophotometrically [33]. The maximum value of the quotient K/S525 and K/S572 at the beginning of the experiment (day 0) was fixed as 0% MMb, while 100% MMb was obtained after oxidizing a beef and pork patties in potassium ferricyanide (1%, w/v). In total, 10 measurements were performed on the surface of each sample, and results were expressed as a percentage of MMb.

### 2.7. Oxidative Stability of Patties

#### 2.7.1. Lipid Oxidation

Lipid oxidation (Lox) was measured by the thiobarbituric acid reactive substances (TBARS) method [34]. Meat samples (10 g) were homogenized with 20 mL of 10% trichloroacetic acid at 4500 rpm for 1 min, in an ice bath. After, the slurry was centrifuged (at 2300× *g*/4 °C/for 20 min) and filtered (Whatman 4 filter paper). In total, 2 mL of filtrate were mixed with 2 mL of TBA (0.02 M) in test tubes, placed in a water bath (97–98 °C) for 20 min, and subsequently cooled. The absorbance was measured at 531 nm. TBARS values were calculated from a 1, 1, 3, 3-tetramethoxypropane standard curve and expressed as mg malondialdehyde/kg of the meat sample (mg MDA/kg).

#### 2.7.2. Protein Oxidation

Protein oxidation (Pox) was measured by the total carbonyl content was evaluated by derivatization with dinitrophenylhydrazine (DNPH) [35]. Meat samples (1 g) were homogenized (1:10, w/v) in 20 mM sodium phosphate buffer containing 0.6 M NaCl (pH 6.5) at 4500 rpm for 1 min. Two equal aliquots of 0.2 mL were taken from homogenates and each dispensed in 2 mL test tubes. Proteins were precipitated with 1 mL of cold TCA (10%, w/v) and subsequently centrifuged at 4200× *g*/4 °C/for 5 min (one pellet was treated with 1 mL of 2 M HCl for protein concentration measurement and the other with an equal volume of DNPH in 2 M HCl for carbonyl concentration measurement). Both samples were incubated at room temperature for 1 h. After, samples were precipitated with 1 mL of TCA (10%, w/v) and washed twice with 1 mL of ethanol:ethyl acetate (1:1, v/v) to remove excess DNPH. Then, each pellet was mixed with 1.5 mL of 20 mM sodium phosphate buffer containing 6 M guanidine HCl (pH 6.5), stirred and centrifuged at 4200× *g*/4 °C/ for 2 min to remove insoluble fragments. Protein concentration was calculated from absorption at 280 nm using BSA as the standard. The amount of carbonyls was expressed as nM of carbonyl per mg of protein using a molar absorption coefficient of 21 nM^−1^ × cm^−1^ at 370 nm for protein hydrazones.

### 2.8. Statistical Analysis

Three independent experimental trials (replications) were conducted, and the results were presented as the mean ± standard deviation. Data of experimental patties were submitted to analysis of variance (ANOVA) according to a two factorial design using NCSS statistical software (2007). Normal distribution and variance homogeneity were previously tested (Shapiro-Wilk). The treatments (B, B+PEE, B+BHT, B+Asc, P, P+PEE, P+BHT, and P+Asc) and storage times (0, 3, 7, and 10 days) were the fixed terms in the model. A Tukey–Kramer multiple comparison test was performed to determine the significance of mean values for multiple comparisons at α ˂ 0.05.

## 3. Results

### 3.1. Polyphenol Composition and Antioxidant Activity

As shown in Table 1, PEE was tested for total phenolic content (TPC) and antioxidant activity (i.e., reducing-power ability, RPA; and, free-radical scavenging activity, FRSA). The results showed that PEE had high TPC (>400 mg GAE/g), RPA (>50% of reduction), and FRSA (>50% of radical inhibition) in concentration-dependence (*p* < 0.05). While the standards (BHT > Asc) showed the highest antioxidant activity (>50%).

In addition, as shown in Table 2, the phenolic compounds identified in PEE were gallic acid, cinnamic acid, p-coumaric acid, naringenin, quercetin, luteolin, kaempferol, apigenin, pinocembrin, pinobanksin 3-acetate, caffeic acid phenethyl ester (CAPE), chrysin, galangin, acacetin, and pinostrobin. However, pinocembrin, naringenin, and galangin were the most abundant flavonoids in the extract (*p* < 0.05).

### 3.2. Total Antioxidant Activity of Meat Extract

**Figure 1:**
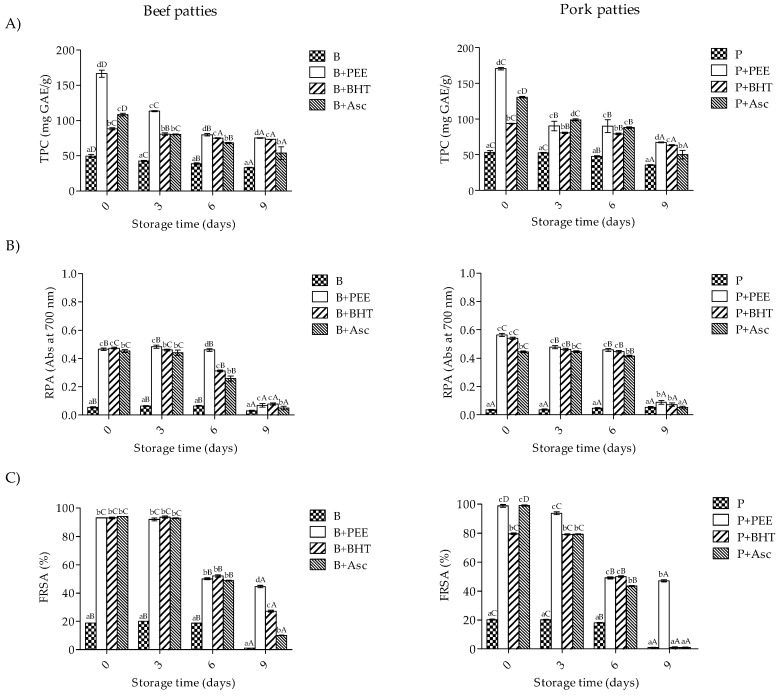
Antioxidant values of beef and pork patties during storage time determined by the total phenolic content (**A**), reducing power ability (RPA) (**B**), and DPPH^●^ radical scavenging activity (FRSA) (**C**). B, beef patties without antioxidant; B+PEE, beef patties with the extract; B+BHT, beef patties with 0.02% of BHT; B+Asc, beef patties with ascorbic acid 0.015%. P, pork patties without antioxidant; P+PEE, pork patties with the extract; P+BHT, pork patties with 0.02% of BHT; P+Asc, pork patties with ascorbic acid 0.015%. Different superscripts within the same sampling day (a–d) and though storage (A–D) differ significantly (*p* < 0.05).

As shown in Figure 1, meat extract from beef and pork patties treated with PEE and synthetic antioxidants were obtained each sampling day and tested for the total antioxidant activity, which was measured through the TPC, RPA, and FRSA. The results showed that treatment × storage time had a significant effect on these parameters (*p* < 0.001). At day 0 of storage, the highest TPC (>160 mg GAE/g for both meat types), RPA (0.56 and 0.47 abs, respectively), and FRSA (98.8% and 93% of radical inhibition, respectively) were found in beef and pork patties treated with PEE, in comparison to synthetic antioxidants and control samples. However, these values significantly decreased during storage time for all treatments (*p* < 0.05). At the end of storage (day 9), beef (55.8%, 54.6%, and 38.2%, respectively) and pork (46.9%, 43.7%, and 28.8%, respectively) patties treated with antioxidants showed higher values (*p* < 0.05) of TPC than the control samples. In this way, the best results were found in the samples treated with PEE, followed by BHT and Asc. Also, beef and pork patties treated with PEE and BHT showed the highest values of RPA (46.3% and 34.8%, respectively), in comparison to the control and Asc treatments (*p* < 0.05). In addition, beef patties treated with PEE had higher FRSA values (*p* < 0.05) than those treated with synthetic antioxidants (97.8%, 96.3%, and 90.3% for PEE, BHT, and Asc, respectively); while, pork samples treated with PEE showed the highest FRSA (97.9%) in comparison to BHT, which displayed similar values to Asc and the control samples (*p* < 0.05).

### 3.3. pH and Oxidation Values of Patties

**Figure 2:**
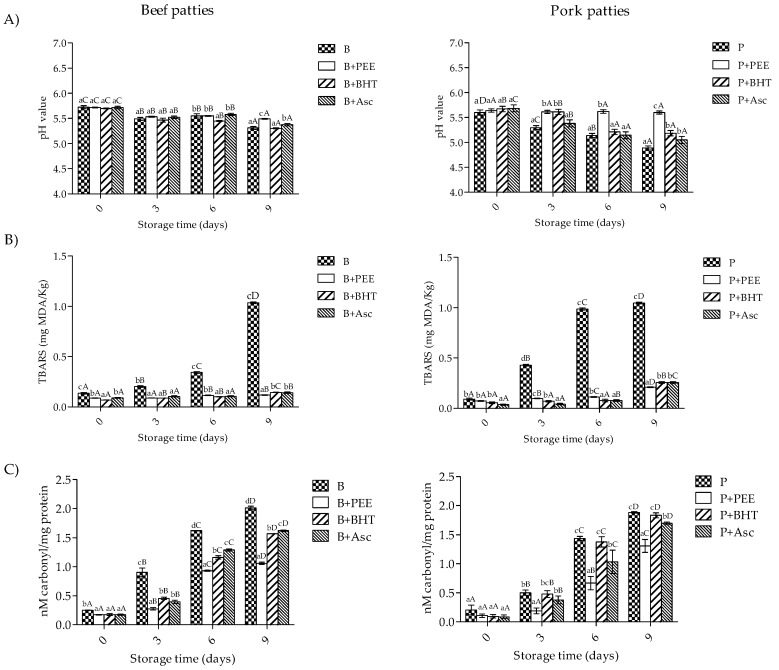
pH (**A**), Lox (RPA) (**B**), and Pox values (**C**) of beef and pork patties during storage time. B, beef patties without antioxidant; B+PEE, beef patties with the extract; B+BHT, beef patties with 0.02% of BHT; B+Asc, beef patties with ascorbic acid 0.015%. P, pork patties without antioxidant; P+PEE, pork patties with the extract; P+BHT, pork patties with 0.02% of BHT; P+Asc, pork patties with ascorbic acid 0.015%. Different superscripts within the same sampling day (a–d) and though storage (A–D) differ significantly (*p* < 0.05).

As shown in Figure 2, beef and pork patties were tested for the pH, Lox, and Pox. The results showed that treatment × storage time had a significant effect on these parameters (*p* < 0.001). At day 0 of storage, the lowest TBARS and carbonyl formation values were found in samples treated with any antioxidant (*p* < 0.05), while no significant differences were shown in pH values (*p* > 0.05). However, these values significantly decreased during storage time (*p* < 0.05). At the end of storage, beef and pork patties treated with PEE showed the highest pH values (5.5 and 5.6, respectively), as well as the lowest meat oxidation values (<0.5 mg MDA/kg; and <1.5 nM carbonyl/mg for both meat types) (*p* < 0.05).

### 3.4. Metamyoglobin Formation of Patties

**Figure 3:**
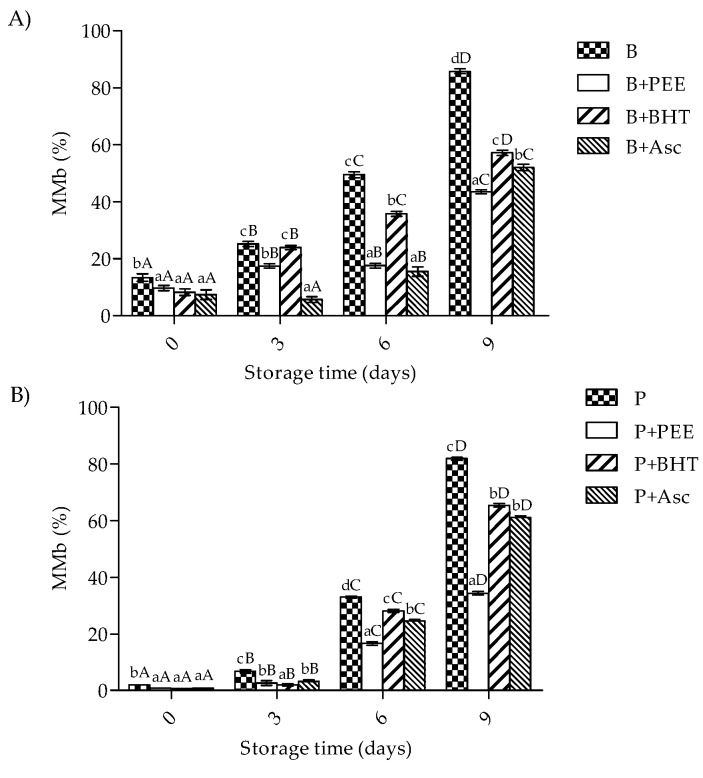
Metmyoglobin formation (MMb) formation in beef (**A**) and pork patties (**B**) during storage time. B, beef patties without antioxidant; B+PEE, beef patties with the extract; B+BHT, beef patties with 0.02% of BHT; B+Asc, beef patties with ascorbic acid 0.015%. P, pork patties without antioxidant; P+PEE, pork patties with the extract; P+BHT, pork patties with 0.02% of BHT; P+Asc, pork patties with ascorbic acid 0.015%. Different superscripts within the same sampling day (a–d) and though storage (A–D) differ significantly (*p* < 0.05).

As shown in Figure 3, beef and pork patties were tested for MMb formation, and results showed that treatment × storage time had a significant (*p* < 0.001) effect on this parameter. At day 0 of storage, the results showed no significant differences in MMb formation in all pork patties (*p* > 0.05), while the highest MMb formation (>10%) was found in beef patties from the control group (*p* < 0.05). In addition, these values significantly increased during storage time (*p* < 0.05). At the end of storage, beef and pork patties treated with PEE showed the lowest MMb content (<40% of MMb for both meat types) (*p* < 0.05).

### 3.5. Color Changes of Patties

As shown in Table 3, beef and pork patties were tested for color change analysis, and the results showed that the treatment × storage time effect was significant (*p* < 0.001). At day 0 of storage, the results of L* values showed no significant effect by antioxidant addition in beef and pork patties (L* = 44.8 and 55.6, respectively). These values significantly increased during storage time (*p* < 0.05). At the end of storage, beef and pork patties treated with PEE and BHT showed the lowest L* values (L* = 45.7 and 57.4, respectively) (*p* < 0.05). In addition, initial a* values showed no significant differences between treatments in beef and pork patties (a* = 22.8 and 20.4, respectively). These values significantly decreased during storage time (*p* < 0.05). At the end of storage, beef and pork patties treated with PEE showed the highest a* values (a* = 15.0 and 15.7, respectively) (*p* < 0.05). While at day 0 of storage, the results showed that patties treated with PEE showed the highest b* values (b* = 20.4 and 21.8, respectively) (*p* < 0.05). These values decreased during storage time (*p* < 0.05). At the end of storage, beef and pork patties treated with PEE showed the highest b* values (b* = 17.5 and 19.1, respectively) (*p* < 0.05). Moreover, at day 0 of storage, the C* and h* values were reduced and increased by PEE addition for both meat type, respectively. At the end of storage, beef and pork patties treated with PEE showed the highest C* values (C* = 23.7 and 24.4, respectively), as well as the lowest h* values (h* = 46.5 and 46.1, respectively) (*p* < 0.05).

## 4. Discussion

Phenolic compounds, one of the major groups found in plants and honeybee products, have been reported to possess antioxidant activity [36]. The Folin–Ciocalteau method is considered the main tool for measuring the TPC and estimating the amount of antioxidant compounds in extracts from different natural sources [26]. The results obtained showed that PEE is an important source of phenolic constituents, such as phenolic acids and their esters and flavonoids. These findings are in agreement with those previously observed by other authors [36]. Whence, PEE was analyzed by HPLC-DAD in order to identify the phenolic composition, and the results showed that flavanones pinocembrin and naringenin, and the flavonol galangin, represent the major (80.1%) compounds quantified. In agreement with our work, it has been reported that pinocembrin and galangin flavonoids are the main phenolic compound in propolis extracts from semiarid regions [25,37].

Phenolic compounds can act in many different ways: 1) reducing metals such as iron (Fe^3+^) and copper (Cu^2+^); and 2) breaking chain reactions triggered by free-radicals, which implies the hydrogen atom transfer (HAT), sequential proton-loss electron-transfer (SPLET) and electron transfer followed by proton transfer mechanism (SET-PT) [38,39]. However, the solvent properties, radical characteristics, and number and position of the hydroxyl group (OH) and first reaction OH-site in the phenolic compound determine these antiradical mechanisms [39]. There are available methods used to assay the RPA and FRSA of natural extracts [27,28]. In our study, PEE showed high RPA and FRSA values (0.56 abs and 69%, respectively) in concentration-dependence, which was significantly correlated with the TPC (*r*^2^ = 0.997). In agreement with our study, it has been reported that PEE collected in Turkey exerted high RPA and FRSA (i.e., >50% of activity) in concentration-dependence [40].

The presence of phenolic compounds in natural extracts is highly correlated with the antioxidant activity, whence the natural extracts incorporation to meat and meat products could be an important strategy to improve oxidative stability during storage [6,41]. It has been reported that meat antioxidant stability could be affected by endogenous and non-enzymatic antioxidants, animal species, animal diet, muscle type, lipid composition, and by the ingredients used in the formulation of meat products (e.g., synthetic antioxidant, chloride salt content, and phenolic content of some species or extract) [42,43]. In this context, the presence of phenolic compounds and antioxidant activity of meat extracts have been measured in raw and meat products through the TPC, RPA, and FRSA methods [43]. In our study, samples treated with PEE showed higher TPC, RPA, and FRSA values than those obtained for control during all storage time. In agreement, this has been demonstrated the presence of TPC in raw reindeer (*Longissimus dorsi* and *Semimembranosus*, 27 and 29 mg GAE/g, respectively), and raw beef meat (*L*. *dorsi*, 17.8 mg GAE/g) untreated with antioxidants [44]. Also, it has been reported that the incorporation of sage essential oil (3%, w/w) on raw and cooked beef and pork meat increased the FRSA (>50% of inhibition by both) during storage (12 days at 4 °C) in comparison to the control [45]. In another work, the incorporation of Lotus rhizome knot and leaf extracts on raw and cooked beef and pork ground meat increased the RPA and FRSA (>50% of activity by both) during storage (10 days at 4 °C), compared to the values obtained for the control [15]. In agreement, it has been reported that the addition of destoned olive cake powder in raw beef patties increased FRSA (17.8%) during 14 days of storage at 4 °C in comparison to the control [46].

In our study, the results of pH, Lox, and Pox values showed a significant treatment × storage time effect on these parameters (*p* < 0.001). The initial average pH values in beef and pork patties (5.7 and 5.5, respectively) remained in the characteristic value for fresh meat [30,47]. However, pH values decreased during storage in beef and porcine samples without antioxidants. In disagreement with our work, no significant differences were found in beef patties treated with PEE (2%, w/w) during storage (8 days at 4 °C) [29]. However, it has been demonstrated that a decrease in the pH value of meat has a great impact on sensory attributes (texture-tenderness, appearance-color, flavor) and the oxidative stability of meat, Lox, and Pox [47,48].

It has been reported that Lox occurs via a free-radical chain reaction that proceeds through three steps (initiation, propagation, and termination) [4]. Lipid hydroperoxides (ROOH) have been identified as primary products of autoxidation, while ROOH decomposition, alcohols, ketones, hydrocarbons, volatile organic acids, aldehydes, among others, known as secondary oxidation products [34,49]. Aldehydic products such as malondialdehyde (MDA), can be measured by the reaction with thiobarbituric acid (TBARS). Thus, it is used commonly as a Lox index for meat products [50,51]. In our study, TBARS values were increased during storage time regardless low temperatures (2 °C); however, at the end of storage, the incorporation of antioxidants decreased Lox in the order PEE > BHT = Asc in beef (88.7% and 86.2%, respectively) and pork patties (80% and 75.5%, respectively) in comparison to control. Also, results have shown that treatments with antioxidants not exceeding the limit value (higher than 0.6 mg MDA/kg) were used for a rancid flavor in a meat product [52]. In addition, it has been reported the potential of another natural source of antioxidants against Lox, like clove extract, which decreased the Lox (>90%) of fresh beef patties during 10 days of storage in comparison to synthetic antioxidant (BHT and Asc) and control samples [53].

In agreement with our study, cured pork sausages were treated with some antioxidants (PEE: ethanol-extracted propolis, 0.4% w/w; WEP: water-extracted propolis, 0.6% w/w; DREEP: dried residue of ethanol-extracted propolis, 0.8% w/w; PS: potassium sorbate, 0.1% w/w) and stored during four weeks at 5, 10, and 20 °C. At the end of storage, the results showed that PEE, WEEP, DREEP, and PS decreased Lox (50%, 59%, 35%, and 91%, respectively) in comparison to the control [54]. While, in another work, PEE reduced Lox (78.5%) in beef patties during storage (8 days at 2 °C), which was associated with the presence of phenolic compounds such as quercetin, pinocembrin, kaempferol, and luteolin [29].

In addition, it has been reported that the hydrophobic and hydrophilic groups of phenolic compounds allowed the molecule to interact with non-lipidic and lipidic components. While, 3-hydroxylation of the C-ring, non-modification of the B-ring, and 5,7-hydroxylation of the A-ring allowed the highest membrane interactivity, follow by 3′,4′-dihydroxylation of the B-ring [55]. Also, it has been demonstrated that flavonols (quercetin and rutin) are more effective than flavanones (hesperetin and naringenin) in reducing MDA formation, which is associated with their ability to donate H-atoms from OH-groups. However, the flavanone naringenin exerted the highest interaction with phospholipidic bilayers [56].

Moreover, the quality of meat and meat products could be affected by Pox, which results in the loss of sulfhydryl groups and the carbonyl compounds formation [3]. Protein carbonyls can be generated by (1) direct oxidation of amino acid (lysine, arginine, and proline) side chains, (2) fragmentation of peptide backbone, (3) reactions with reducing sugars, and (4) binding non-protein carbonyl compounds [57]. In our study, Pox values increased during storage regardless of low temperatures. At the end of storage, the incorporation of antioxidants decreased Pox in beef patties (47.3%, 21.9%, and 19.4% for PEE, BHT, and Asc, respectively). In the same way, the incorporation of antioxidants reduced Pox in pork samples, with samples treated with PEE showing the higher reduction values, followed by Asc and BHT (30.6%, 10%, and 2.7%, respectively). In agreement, black currant extract (2%) and BHT (0.2%) addition to pork patties decreased Pox during storage (9 days at 4 °C) in a dose-dependent manner in comparison to the control [58]. In addition, it has been reported that the highest effectiveness of natural extracts against Pox (e.g., curry, cocoa, green tea, oregano, peanut, rosemary, olive, grape, avocado, and broccoli) are rich in phenolic compounds, which may be associated with the faster occurrence of Lox than Pox, and to the interaction between flavonoids and proteins [59,60,61]. In our study, a positive correlation (*r*^2^ = 0.83) was found between Lox and Pox, which demonstrated that both are a timely couple in meat samples [3].

The phenolic compounds identified in PEE (e.g., gallic acid and some flavonols) have been described as inhibitors of carbonyl compound formation from myofibrillar proteins [62]. Additionally, it has been reported that ferulic acid, malvidin, and rutin reduced, in vitro, the losses of tryptophan and lysine associated with Pox [63]. In this context, our results showed that the incorporation of PEE reduced Pox, which could be associated with the phenolic compound–protein interaction mechanisms and correlated to the hydrophobicity of the aromatic ring of polyphenols and to their OH-groups allowing the H-bonding [13,57].

In meat and meat products, color influences the acceptability and plays a great role in the purchase decision, as consumers relate color with sensory attributes (flavor and tenderness) and another’s parameters, such as nutritional value, satisfaction level and storage time [64]. In our study, the initial L* and a* values of beef and pork samples were not affected by PEE incorporation, except for b*, C, and h angle. At the end of storage, beef and pork patties with PEE showed lower L* (1.7 and 3.0%, respectively) and h* values (22.1 and 13.4%, respectively), as well as the higher a* (38.7 and 26.1%, respectively), b* (28 and 15.7%, respectively), and C* values (33.8 and 21.7%, respectively) than those obtained in control samples. In agreement, PEE increased the red color of beef patties during storage 19.4% (8 days at 2 °C) in comparison to the control [29].

Furthermore, the discoloration observed in the surface of raw patties is associated with metmyoglobin formation (MMb), which in fresh meat myoglobin (Mb) is commonly found in three forms: oxymyoglobin [oxy(Fe^2+^)Mb], deoxymyoglobin [deoxy(Fe^2+^)Mb], and metmyoglobin, [met(Fe^3+^)Mb)] [65]. In our work, MMb values increased during storage time regardless of low temperatures; however, at the end of storage, the incorporation of antioxidants decreased MMb in the order PEE > BHT > Asc in beef (49.3%, 33.4%, and 39.4%, respectively). A similar behavior was also found in porcine patties where the incorporation of antioxidants decreased MMb, with the samples treated with PEE showing the largest decreases, followed by samples treated with Asc and BHT (58.1%, 25.3%, and 20.1%, respectively) in comparison to the control.

The MMb values found for PEE during storage are below the limit values (above 40%) that could be associated with decreases in sensory attributes of meat and meat products, and therefore, could affect the consumer purchase, rejecting the product [66]. In agreement, PEE reduced 58.6% MMb in beef patties during storage (8 days at 2 °C) compared to the control samples [29]. Other phenolic-rich extracts, such as tea extracts, seaweed, chestnut, grape seed, kimchi, lycopene, onion, oregano, pine bark, plum, red pepper, rice fiber, and rosemary, have also reported reduced color changes and MMb formation in meat samples during chilled storage [67,68,69,70]. Thus, our study results confirm the efficacy of PEE against Lox and Pox and color changes during chilled storage.

## 5. Conclusions

In this study, the current findings demonstrated an interesting phenolic composition of PEE, as well as high RPA and FRSA. Additionally, the addition of PEE (2%) to raw beef and pork patties reduced Lox and Pox effectively, as well as the color changes that occur during the storage of meat (2 °C/under dark/for 10 days), in comparison with the synthetic antioxidants (BHT and Asc) and the control meat samples (without antioxidant). This study demonstrated great potential for PEE as a promising natural antioxidant for the food industry, increasing the shelf life of meat products, even in patties most susceptible to oxidation. However, further studies on the relationships between lipid and protein oxidation with sensorial quality in beef and porcine patties would need to be carried out in order to corroborate these promising outcomes since MMb percentages lower than 40% would be related to obtaining more attractive products for the consumers. Moreover, novel technologies, such as the active packaging and the microencapsulation, should be addressed to incorporate propolis extract on meat products, as well as a microbiological evaluation, which would allow the assessment of antimicrobial activity of this extract.

## Figures and Tables

**Table 1:** Total phenolic content and antioxidant activity of propolis ethanol extract (PEE).

(µg/mL)	TPC (mg GAE/g)	RPA (Abs)	FRSA (%)
500	472.3 ± 3.50 ^f^	0.56 ± 0.03 ^g^	69.1 ± 0.03 ^e^
250	288.1 ± 0.53 ^e^	0.34 ± 0.01 ^e^	45.7 ± 0.11 ^d^
100	198.5 ± 0.98 ^d^	0.20 ± 0.01 ^d^	33.0 ± 0.01 ^c^
50	152.5 ± 1.22 ^c^	0.14 ± 0.01 ^c^	31.0 ± 0.05 ^b^
25	126.6 ± 0.24 ^b^	0.10 ± 0.01 ^b^	30.0 ± 0.01 ^b^
12.5	122.9 ± 0.53 ^a^	0.07 ± 0.01 ^a^	28.7 ± 0.01 ^a^
BHT (50 µg/mL)	ND	0.7 ± 0.01 ^h^	70.8 ± 0.10 ^e^
Asc (25 µg/mL)	ND	1.0 ± 0.01 ^i^	73.0 ± 0.10 ^f^

ND: not determined. Data represent mean ± standard deviation. Different superscripts (a–i) within the same column differ significantly (*p* < 0.05).

**Table 2:** Phenolic compounds identified in PEE by ethanol and methanol diode array detector (HPLC-DAD).

#	Compound	Retention Time (min)	PEE (mg/g) ^a^
1	Gallic acid	1.9	(+)
2	Cinnamic acid	3.4	2.1 ± 0.2 ^b^
3	*p*-coumaric acid	7.8	2.9 ± 0.1 ^c^
4	Ferulic acid	8.7	(−)
5	Naringenin	27.3	50.2 ± 5.9 ^j^
6	Quercetin	30.8	6.5 ± 0.2 ^f^
7	Luteolin	36.4	3.7 ± 0.2 ^d^
8	Kaempferol	37.2	0.9 ± 0.2 ^a^
9	Apigenin	40.6	4.4 ± 0.2 ^e^
10	Pinocembrin	44.5	130.7 ±1.8 ^k^
11	Pinobanksin 3-acetate	45.8	(+)
12	CAPE	49.0	(+)
13	Chrysin	51.4	12.3 ± 1.0 ^h^
14	Galangin	52.4	37.0 ± 2.1 ^i^
15	Acacetin	57.0	8.4 ±0.4 ^g^
16	Pinostrobin	62.9	(+)

(+), compound identified but not quantified; (−), compound not identified. Data represent mean ± standard deviation. Different superscripts (a–k) within the same column differ significantly (*p* < 0.05).

**Table 3:** Instrumental color measured during storage time on the surface of beef and pork patties.

Item		Beef				Pork			
	Day	B	B+PEE	B+BHT	B+Asc	P	P+PEE	P+BHT	P+Asc
L*	0	44.0 ± 0.9 ^aA^	45.0 ± 0.6 ^aA^	45.5 ± 0.6 ^aA^	44.7 ± 0.6 ^aA^	54.2 ± 1.3 ^aA^	56.2 ± 0.8 ^aA^	56.4 ± 0.9 ^aA^	55.6 ± 0.9 ^aA^
	3	43.1 ± 0.8 ^aA^	44.7 ± 0.7 ^bA^	45.7 ± 0.6 ^bA^	44.6 ± 0.4 ^bA^	53.1 ± 1.5 ^aA^	57.2 ± 0.8 ^bA^	56.8 ± 1.0 ^bA^	56.8 ± 0.7 ^bA^
	6	44.1 ± 0.7 ^aA^	46.3 ± 0.3 ^cB^	45.4 ± 0.6 ^bA^	45.3 ± 0.2 ^bA^	56.1 ± 1.0 ^aA^	57.0 ± 0.3 ^aA^	57.5 ± 1.0 ^aA^	59.7 ± 0.7 ^bB^
	9	47.0 ± 0.5 ^bB^	46.2 ± 0.5 ^aB^	45.1 ± 0.6 ^aA^	47.5 ± 0.1 ^bB^	59.4 ± 1.5 ^bB^	57.6 ± 1.0 ^aA^	57.2 ± 0.8 ^aA^	59.9 ± 0.9 ^bB^
a*	0	23.2 ± 0.6 ^aD^	22.1 ± 0.4 ^aC^	23.0 ± 0.4 ^aD^	23.0 ± 1.0 ^aC^	20.9 ± 0.5 ^aC^	20.4 ± 0.6 ^aC^	20.2 ± 0.5 ^aC^	19.9 ± 0.6 ^aC^
	3	20.9 ± 0.6 ^bC^	18.4 ± 0.4 ^aB^	20.5 ± 0.4 ^bC^	22.2 ± 0.8 ^cC^	20.0 ± 0.4 ^bC^	17.4 ± 0.6 ^aB^	19.8 ± 0.5 ^bC^	19.6 ± 0.6 ^bC^
	6	11.3 ± 0.6 ^aB^	16.0 ± 0.4 ^bA^	16.9 ± 0.4 ^bB^	19.5 ± 0.7 ^cB^	15.3 ± 0.4 ^aB^	15.8 ± 0.6 ^aA^	17.0 ± 0.5 ^bB^	16.1 ± 0.6 ^bB^
	9	9.2 ± 0.6 ^aA^	15.0 ± 0.4 ^bA^	8.1 ± 0.6 ^aA^	9.5 ± 0.8 ^aA^	11.6 ± 0.6 ^aA^	15.7 ± 0.7 ^bA^	11.1 ± 0.8 ^aA^	10.2 ± 0.6 ^aA^
b*	0	18.3 ± 0.5 ^aC^	20.4 ± 0.5 ^bB^	18.5 ± 0.5 ^aB^	18.2 ± 0.3 ^aC^	17.1 ± 0.6 ^aAB^	21.8 ± 0.4 ^bB^	18.8 ± 0.4 ^aB^	17.6 ± 0.5 ^aB^
	3	16.3 ± 0.5 ^aB^	17.6 ± 0.4 ^bA^	17.3 ± 0.5 ^bB^	18.1 ± 0.6 ^bC^	17.5 ± 0.7 ^aB^	19.7 ± 0.4 ^bA^	19.0 ± 0.4 ^bB^	17.9 ± 0.5 ^aB^
	6	12.6 ± 0.5 ^aA^	17.4 ± 0.4 ^cA^	15.0 ± 0.5 ^bA^	15.3 ± 0.6 ^bB^	16.1 ± 0.5 ^aA^	19.9 ± 0.4 ^cA^	17.4 ± 0.4 ^bA^	17.7 ± 0.5 ^bB^
	9	12.6 ± 0.5 ^aA^	17.5 ± 0.5 ^cA^	14.3 ± 0.6 ^bA^	13.9 ± 0.6 ^bA^	16.1 ± 0.5 ^aA^	19.1 ± 0.4 ^bA^	16.5 ± 0.5 ^aA^	15.9 ± 0.5 ^aA^
C*	0	30.5 ± 0.6 ^bD^	28.7 ± 0.4 ^aC^	30.4 ± 0.5 ^bD^	30.3 ± 0.9 ^bC^	27.5 ± 0.3 ^bC^	26.2 ± 0.4 ^aC^	27.8 ± 0.6 ^bC^	27.4 ± 0.1 ^bD^
	3	27.0 ± 0.6 ^aC^	26.2 ± 0.5 ^aB^	26.9 ± 0.5 ^aC^	28.9 ± 0.9 ^aC^	27.2 ± 0.6 ^bC^	27.5 ± 0.6 ^bB^	26.6 ± 0.6 ^bC^	25.8 ± 0.5 ^aC^
	6	17.0 ± 0.6 ^aB^	24.1 ± 0.5 ^bA^	23.3 ± 0.5 ^bB^	24.8 ± 0.7 ^bB^	23.1 ± 0.6 ^aB^	26.0 ± 0.6 ^bB^	25.0 ± 0.6 ^bB^	23.8 ± 0.5 ^aB^
	9	15.7 ± 0.6 ^aA^	23.7 ± 0.5 ^bA^	16.0 ± 0.7 ^aA^	17.3 ± 0.8 ^aA^	19.1 ± 1.1 ^aA^	24.4 ± 0.5 ^cA^	20.3 ± 0.7 ^bA^	18.7 ± 0.6 ^aA^
h*	0	38.3 ± 0.5 ^aA^	41.9 ± 0.5 ^bA^	38.9 ± 0.4 ^aA^	37.8 ± 0.5 ^aA^	41.7 ± 0.7 ^aA^	43.5 ± 0.5 ^bA^	42.0 ± 0.6 ^aA^	41.2 ± 0.8 ^aA^
	3	38.0 ± 0.5 ^aA^	43.7 ± 0.5 ^cB^	40.2 ± 0.4 ^bB^	37.2 ± 0.5 ^aA^	42.1 ± 0.5 ^aA^	47.0 ± 0.6 ^bB^	43.8 ± 0.6 ^aA^	43.1 ± 0.7 ^aA^
	6	54.8 ± 0.7 ^dB^	46.5 ± 0.6 ^cC^	40.5 ± 0.5 ^bB^	38.2 ± 0.5 ^aA^	44.6 ± 0.5 ^aB^	49.2 ± 0.5 ^cC^	46.1 ± 0.6 ^bB^	48.5 ± 0.7 ^cB^
	9	61.2 ± 0.5 ^cC^	47.7 ± 0.7 ^aC^	59.7 ± 0.6 ^bC^	59.3 ± 0.7 ^bB^	55.9 ± 0.6 ^bC^	48.4 ± 0.6 ^aC^	55.6 ± 0.9 ^bC^	56.2 ± 1.3 ^bC^

Data represent mean ± standard deviation. B, beef patties without antioxidant; B+PEE, beef patties with the extract; B+BHT, beef patties with 0.02% of BHT; B+Asc, beef patties with ascorbic acid 0.015%. P, pork patties without antioxidant; P+PEE, pork patties with the extract; P+BHT, pork patties with 0.02% of BHT; P+Asc, pork patties with ascorbic acid 0.015%. Different superscripts within the same sampling day (a–d) and though storage (A–D) differ significantly (*p* < 0.05).
